# Non infective severe aortic paravalvular leakage 7 years after surgery: the role of suture technique

**DOI:** 10.1186/1749-8090-6-60

**Published:** 2011-04-23

**Authors:** Marco Agrifoglio, Sara Filippini, Maurizio Roberto, Marco Zanobini, Luisa Gregorini, Francesco Alamanni

**Affiliations:** 1 Department of Cardiovascular Sciences, Centro Cardiologico Monzino IRCCS, University of Milan, Via C. Parea 4, 20138 Milan, Italy

**Keywords:** aortic valve replacement, paravalvular leakage, suture technique

## Abstract

We report a case of redo aortic prosthesis replacement for a severe paravalvular leak (PVL) in a man operated with continuous suture technique 7 years earlier. The severe aortic regurgitation was due to the rupture of the suture. In spite of operations to replace malfunctioning heart valves are common procedures and performed all over the world from more than 50 years, there is still an open debate about the most suitable suture technique. In this case report, we'll discuss if the suture technique has a role in preventing or leading complications as severe PVL.

## Introduction

Paravalvular leak (PVL) after valve replacement is a rare, but serious complication. Most frequently PVL is caused by a prosthetic valve endocarditis, but it may also occurs without definite signs of infection. We report a case of redo aortic prosthesis replacement after 7 years from the operation and we discuss the role of suture technique in this case of severe paraprosthetic leak without any clearly infection signs.

## Case Report

A 48-year-old man came to our hospital for sudden effort dyspnea. The patient underwent seven years ago to aortic valve replacement (AVR) with 25 mm On-X (On-X Life Technology Inc., Austin, TX, USA) mechanical prosthesis for severe aortic valve regurgitation and ascending aorta replacement with a tube graft n 28 for ascending aorta aneurysm. The AVR was performed using three semicontinuous (2-0 Prolene, Ethicon, Somerville, NJ, USA) suture technique. The surgeon used three similar sutures with knots tied among them, hence the term semicontinuous. According to a "parachute technique" the sutures were suspended (left loose) to enable an easier suturing of the aortic annulus and sewing ring of the artificial valve prior to lower the prosthesis. Then a gentle alternate traction on the three semicontinuous sutures, usually with the help of a surgical nerve hook, is applied to pull up the redundant suture. This could weaken the suture and lead to its failure at a later date.

**Figure 1:**
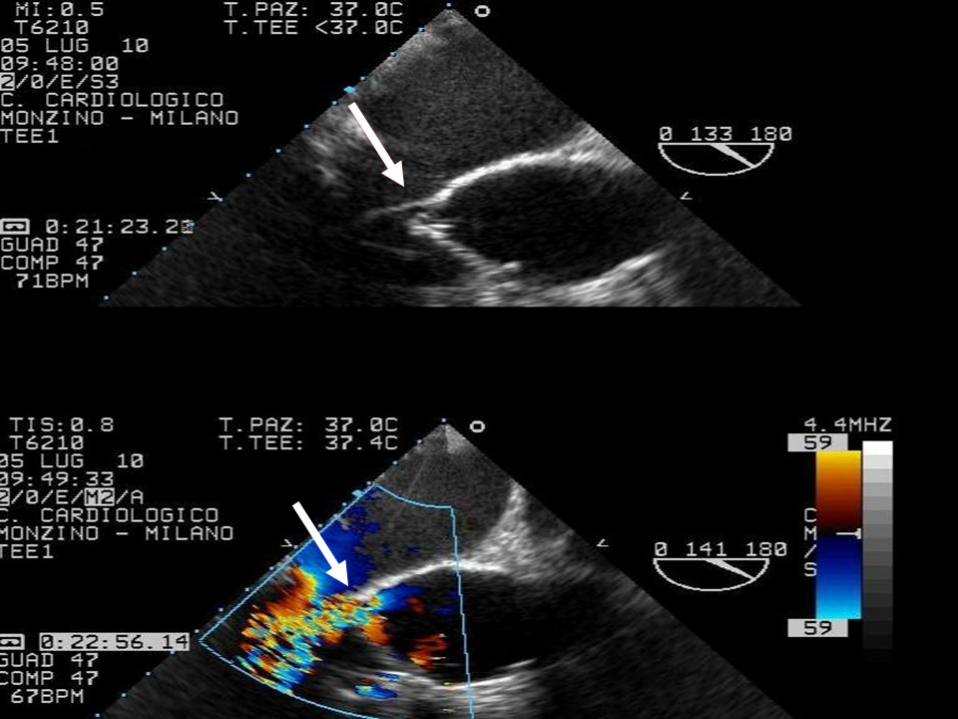
**The transesophageal echocardiogram shows severe aortic prosthetic regurgitation (white arrow), due to significant paravalvular leakage in the non-coronary aortic sinus**.

A yearly transthoracic echocardiography (TTE) follow-up didn't show any pathological findings. The TTE, performed as soon as the patient was admitted to Emergency Room (ER), revealed a severe aortic regurgitation due to a PVL. The ejection fraction was normal but the left ventricle was dilated: indexed end diastolic volume (iEDV) 107 ml, indexed end systolic volume (iESV) 45 ml. The transesophageal echocardiogram (TEE) confirmed the severe aortic regurgitation, showing an aortic non coronary sinus PVL with a partial detachment of aortic prosthesis. Figure 1 clearly documents the PVL.

Preoperative 3 D, 24 slices, Computed Tomography Scan (CT-scan) showed perfect patency of coronary arteries and severe retrosternal adhesions.

**Figure 2:**
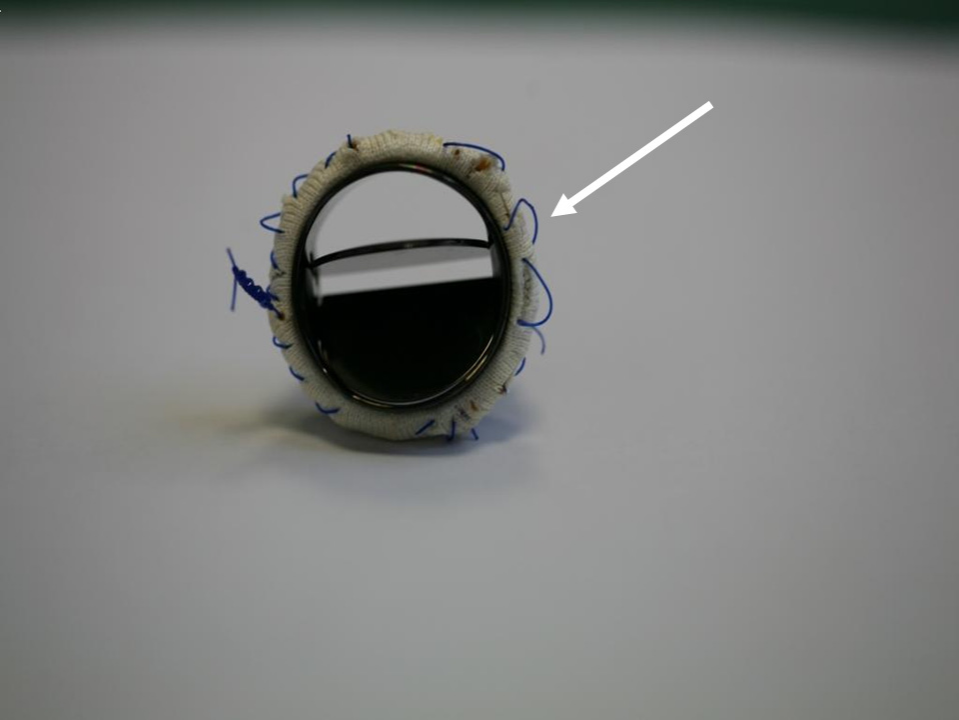
**The aortic mechanical prosthesis shows a partial detachment at the level of the non-coronary aortic sinus**. The semicontinuous suture was broken (white arrow) and the knot was loose. In the picture only one knot is visible since the last one was cut to make possible the aortic prosthesis explant.

After the sternum reopening and adhesions dissection, the ascending aorta tube graft was opened. The surgical findings confirmed a partial prosthesis detachment at the level of the non coronary aortic sinus, due to the semicontinuous suture breaking (Figure 2). The other two sutures of the left and right coronary sinuses were intact. After the prosthesis explant, a new aortic mechanical prosthesis was implanted (23 mm S. Jude, S. Jude Medical, St. Paul, MN, USA), using interrupted suture technique with 2-0 Ethibond (Ethicon) polytetrafluoroethylene (Teflon)-buttressed stitches. The Teflon buttresses were positioned in the ventricular aspect of the annulus. The postoperative TEE showed normal position and function of the prosthesis The postoperative course was uneventful and the patient was shortly discharged.

## Discussion

AVR can be performed using either the semicontinuous or interrupted suture technique depending on the surgeons' choice. The undesired complication of PVL after AVR is a serious complication that can lead to heart failure, thromboembolic and hemolytic complications.

After valve replacement the factors that may mainly favor PVL formation are the annulus calcification and infections.

We report a case of paraprosthetic leakage, occurring seven years after AVR using a continuous suture technique. There are some controversies about the incidence of PVL with continuous suture technique. From one hand Englberger et al. reported an incidence of PVL of 5,8% [1], Hyelms et al. reported of 8,8% [2], Nair et al. even 12% in patients in whom a continuous suture was used [3]. On the other hand Laks et coworkers [4] had incidence of PVL of only 2.3% and Qicai [5] surprisingly 0%. The advantages of the semicontinuous suture technique may consist in a shorter CPB, in a reduced myocardial ischemic injury time, and in shorter cross-clamping time, that may be of benefit in patients with poor left ventricular function. A shorter recovery could streghten the patients and may reduce also the occurrence of endocarditis after prosthetic valve implantation. An additional advantage of the continuous suture technique may be the presence of less thrombogenic material (pledgettes) around the prosthesis [5].

The advantages of interrupted sutures: you can avoid a complete, and sometimes, very dangerous decalcification, expecially when calcium is very close to coronary ostia or very deep in the ventricular septum or involving the anterior leaflet of mitral valve; the supraannular position permits a prosthesis oversizing to reduce the risk of patient mismatch, without increasing the risk of PVL.

At present TTE or TEE are now widely performed in patients after heart valve replacement. Recently three dimensional (3D) TTE has been introduced as non invasive tool reducing the need of TEE to describe and localize PVL. Actually, at this moment, it should be considered still complementary to two dimensional (2D) TTE and TEE [6,7].

Small and asymptomatic paraprosthetic jets are often detected incidentally as a result of high sensitivity color flow mapping [8]. These small leaks are at short term usually benign, but they require an accurate long-term follow-up for an early detection of a major paraprosthetic regurgitation that needs a second intervention.

## Conclusions

Despite the annual aortic echocardiographic monitoring our patient had a sudden onset of dyspnea and a rapid cardiac failure caused by a non expected suture rupture.

For this reason an interrupted suture technique, as we usually do in our Department, could be the best choice to avoid a sudden, severe and sometimes life threatening complication as the rupture of almost one third of annulus suture line.
